# JNK signaling mediates acute rejection via activating autophagy of CD8^+^ T cells after liver transplantation in rats

**DOI:** 10.3389/fimmu.2024.1359859

**Published:** 2024-03-18

**Authors:** Xiaowen Wang, Wenfeng Zhu, Haoqi Chen, Xuejiao Li, Wenjie Zheng, Yuan Zhang, Ning Fan, Xiaolong Chen, Genshu Wang

**Affiliations:** ^1^ Department of Hepatic Surgery, Liver Transplantation, The Third Affiliated Hospital of Sun Yat-Sen University, Guangzhou, China; ^2^ Guangdong Key Laboratory of Liver Disease Research, The Third Affiliated Hospital of Sun Yat-Sen University, Guangzhou, China; ^3^ State Key Laboratory of Traditional Chinese Medicine Syndrome, Guangdong Provincial Hospital of Chinese Medicine, The Second Affiliated Hospital of Guangzhou University of Chinese Medicine, Guangzhou, China; ^4^ Department of Hepatobiliary Surgery, The First Affiliated Hospital of Jinan University, Guangzhou, China

**Keywords:** JNK signaling, autophagy, CD8^+^ T cells, acute rejection, liver transplantation

## Abstract

**Background:**

Acute rejection (AR) after liver transplantation (LT) remains an important factor affecting the prognosis of patients. CD8^+^ T cells are considered to be important regulatory T lymphocytes involved in AR after LT. Our previous study confirmed that autophagy mediated AR by promoting activation and proliferation of CD8^+^ T cells. However, the underlying mechanisms regulating autophagy in CD8^+^ T cells during AR remain unclear.

**Methods:**

Human liver biopsy specimens of AR after orthotopic LT were collected to assess the relationship between JNK and CD8^+^ T cells autophagy. The effect of JNK inhibition on CD8^+^ T cells autophagy and its role in AR were further examined in rats. Besides, the underlying mechanisms how JNK regulated the autophagy of CD8^+^ T cells were further explored.

**Results:**

The expression of JNK is positive correlated with the autophagy level of CD8^+^ T cells in AR patients. And similar findings were obtained in rats after LT. Further, JNK inhibitor remarkably inhibited the autophagy of CD8^+^ T cells in rat LT recipients. In addition, administration of JNK inhibitor significantly attenuated AR injury by promoting the apoptosis and downregulating the function of CD8^+^ T cells. Mechanistically, JNK may activate the autophagy of CD8^+^ T cells through upregulating BECN1 by inhibiting the formation of Bcl-2/BECN1 complex.

**Conclusion:**

JNK signaling promoted CD8^+^ T cells autophagy to mediate AR after LT, providing a theoretical basis for finding new drug targets for the prevention and treatment of AR after LT.

## Introduction

Liver transplantation (LT) remains the only effective treatment for end-stage liver disease. The improvement of surgical procedure and immunosuppressants significantly improve the prognosis of LT recipients. However, acute rejection (AR) after LT still seriously affects the quality of life and long-term survival of patients. In the current usage of powerful immunosuppressants such as cyclosporine A and tacrolimus, the incidence of AR after LT remains as high as 15.6%-26.9% (1). In addition, liver and kidney toxicity and infection risk caused by immunosuppressive agents should not be ignored (2–4). Therefore, it is of great clinical significance to study the mechanism of rejection after LT and to find new drug targets for the prevention and treatment of AR.

AR after liver transplantation is mainly mediated by T cells, also known as T-cell-mediated rejection (TCMR) (5, 6). Among which, CD4^+^ T cells promote parenchymal cell apoptosis through Fas-FasL, attract and recruit neutrophils and macrophages to damage graft by secreting inflammatory cytokines, and help activate CD8^+^ T cells. Activated CD8^+^ T cells can kill graft and vascular endothelial cells directly by secreting granzyme and perforin (7, 8). Therefore, inhibition of T lymphocyte proliferation and promotion of T lymphocyte apoptosis are important means to induce immune tolerance. Our previous study has proved that the activated CD8^+^ T cells during AR rely on autophagy to promote their proliferation and function, and the inhibition of autophagy prolongs rat recipient survival by promoting the apoptosis of CD8^+^ T cells (9). Our previous study indicated that manipulate autophagy may be effective strategy to induce immune tolerance. However, the underling mechanism of CD8^+^ T cell autophagy participates in AR after LT have not been previously investigated.

JNK is a member of the mitogen-activated protein kinase (MAPK) family, located in the c-Jun amino terminal activation region, and consists of three subtypes: JNK1, JNK2, and JNK3. JNK1 and JNK2 subtypes are widely expressed in various tissues and organs, while JNK3 is expressed mainly in the brain and nerves (10). The JNK pathway is activated in response to a variety of stimuli, such as oxidative stress, inflammation, DNA damage, and cytoskeleton remodeling (11–13). JNK pathways are reported to promote the expression of various autophagy related genes including Atg5, Atg7 and Beclin-1 directly or through FoxOs, thereby activating autophagy (14). Besides, JNK signaling plays an important role in T cell activation and proliferation (15, 16). And the role of JNK activation in transplantation immunity has also been explored by some researchers. Inhibition of JNK was found to suppress T cell-mediated immune response and attenuate AR after heart and kidney transplantation (17, 18). However, whether JNK signaling pathway can mediate hepatic AR by promoting proliferation of CD8^+^ T cells through up-regulation of autophagy remains to be further elucidated.

In the present study, we collected sixteen AR patients’ liver sections and confirmed the positive correlation between the JNK expression and the autophagy level of CD8^+^ T cells. And similar findings were obtained *in vivo* by establishing liver transplantation model in rats. Further, JNK inhibitor remarkably inhibited the autophagy of CD8^+^ T cells in rat LT recipients. In addition, administration of JNK inhibitor significantly attenuated AR injury by promoting the apoptosis and downregulating the function of CD8^+^ T cells. Mechanistically, JNK may activate the autophagy of CD8^+^ T cells through upregulating BECN1 by inhibiting the formation of Bcl-2/BECN1 complex. Finally, we further demonstrated that JNK signaling up-regulates BECN1-promoted autophagy mediated AR after LT in a rat model. In conclusion, our results indicate that inhibition of JNK signaling can be used as one of the means to target autophagy of CD8^+^ T cells and might become a new strategy for the treatment graft rejection.

## Materials and methods

### Clinical liver samples and liver function tests

16 paraffin-embedded liver sections of human liver tissue with different grades of rejection after LT and 10 control sections with normal liver histology from hepatic hemangioma patients were obtained from the Department of Pathology of the Third Affiliated Hospital of Sun Yat-Sen University. The diagnosis of AR was according to Banff criteria (19). The sections were used for histological and immunostaining analysis. Informed consent was obtained from all the patients in accordance with the ethics committee of the Third Affiliated Hospital of Sun Yat-Sen University.

### Hepatic acute rejection model in rats

Specific pathogen-free male Lewis (LW) and male Brown Norway (BN) rats (8 to 12 weeks old, 200 to 250 g in weight) were purchased from Beijing Vital River Company. All rats were housed at the Institute of Laboratory Animal Science, Jinan University, at 21°C and under a 12/12-hour light/dark cycle with ad libitum access to sterile water and standard pellet chow under standardized environmental conditions. An orthotopic liver transplantation (OLT) model without hepatic artery reconstruction was created as our previous studies described (9, 20). Rats were randomly divided into two groups: homogenic group and allogenic group. In allogenic group, acute rejection model was established by transplanting LW livers into BN rats. And transplantation model from BN to BN was used as homogenic control. In some experiments, allogenic rat recipients were treated with JNK inhibitor SP600125 intraperitoneally at a dose of 10mg/kg every 2 days starting from the day before transplantation. The rats in each group were sacrificed on days 3, 7, and 9 after LT according to experimental design via anesthesia overdose, respectively. Before the rats were sacrificed, serum samples were obtained. All procedures complied with the “Guide for the Care and Use of Laboratory Animals” from the National Institutes of Health (Approval numbers: RGBIO 2022091601).

### Isolation of lymphocytes and cell culture

Peripheral blood mononuclear cells (PBMCs) were isolated from the peripheral blood of patients and rat recipients by Ficoll density-gradient centrifugation according to the manufacturer’s instructions. PBMCs were plated in 96-well round-bottom plates in RPMI-1640 complete medium (RPMI-1640 medium (Invitrogen, USA), 10% FBS (Gibco, USA) and 1% penicillin/streptomycin (Invitrogen, USA)) at 37°C and 5% CO_2_. Rat one-way mixed lymphocyte reaction (MLR) model was established. Briefly, donor (LW rat) lymphocytes were inactivated by mitomycin C (final concentration 25 μg/mL) at 37 °C for 30 min, washed by PBS for 3 times, and resuspended in RPMI-1640 complete medium. The lymphocytes of recipient (BN rat) also were resuspended in RPMI-1640 complete medium for standby. The density of donor and recipient cells was adjusted to 1×10^6^/mL, and 100μL of donor and recipient cells were added into a 96-well plate with a round bottom (1:1 ratio), with a total volume of 200μL. CD8^+^ T cells were isolated by magnetic affinity cell sorting using CD8 microbeads (MiltenyiBiotec, Germany). To confirm the purification, CD8^+^ T cells were stained with BV510-conjugated anti-CD3 and APC-conjugated anti-CD8, and then assayed by flow cytometry (**Supplementary Figure 1A**). Jurkat T cells (American Tissue Culture Collection, USA) were cultured under the same conditions as PBMCs.

### Cell transfection and treatment

Plasmid and siRNA were used to regulate the expression of JNK and BECN1. JNK-siRNA (RiboBio, China), JNK-overexpressing and BECN1-shRNA plasmid (GeneCopoeia, USA) were transfected using Lipofectamine 3000 (Invitrogen, USA) according to the manufacturer’s instructions. All transfections were independently performed at least three times. Jurkat cells are activated by anti-CD3/28 mAbs (Invitrogen, USA). We established a stable expression stubRFP-sensGFP-LC3 Jurkat T cell line infected by directly adding lentivirus expressing stubRFP-sensGFP-LC3 fusion protein (GeneChem, China) following the manufacturer’s protocol. Jurkat cells and CD8^+^ T cells were treated with or without 3 mM 3-methyladenine (3-MA, Sigma-Aldrich, USA) for 2 days. For SP600125 (Selleck Chemicals, USA) treatment, cells were incubated with or without SP600125 for 3 days at a dose of 5 μM.

### Depletion of CD8^+^T cells by antibodies and CD8^+^ T cells intravenous injections

The recipient BN rats were injected with anti-rat CD8 antibody (Novus, OX-8, 0.5 mg/Rat) through the tail vein three days before liver transplantation, while the control group was injected with CD8 immunoglobulin G (IgG). The proportion of CD8^+^ T cells in liver tissue and peripheral blood was detected at different time points after injection to observe the effect of CD8^+^ T cells depletion. In addition, CD8^+^ T cells were isolated from the spleen of recipient BN rats using a magnetic CD8^+^ T cells isolation kit (MiltenyiBiotec, Germany). A small fraction of the collected cells was used to ensure the purity of the isolated CD8^+^ T cells by flow cytometry. The cells were resuspended in PBS solution immediately before injection, and 3.2 × 10^6^ CD8^+^ T cells were injected into homologous recipient BN rats through the tail vein before liver transplantation.

### Liver histological examination and immunohistochemical staining

Liver tissues were fixed in 10% paraformaldehyde, embedded in paraffin and then cut into sections in 4mm thickness. Liver tissues sections were stained with hematoxylin and eosin (H&E) for histological examination using standard histological procedures. The degree of AR was assessed by two pathologists using the Banff scheme to calculate the rejection activity index (RAI) based on three individual characteristics (venous endothelial inflammation, bile duct damage and portal inflammation). For immunohistochemical staining, after dewaxed, hydrated, antigen repaired, the sections were incubated with corresponding primary antibodies overnight at 4°C, including p-JNK(Cell Signaling Technology, CST, USA), CD8 (CST, USA) and LC3B (ABclonal, China). After washed twice with PBS, the sections incubated with HRP-linked secondary antibody (DAKO, USA) for 1 h at room temperature. At last, the sections were visualized using DAB (DAKO, USA) and counterstained using hematoxylin.

### Immunofluorescence triple-labeling of CD8, p-JNK and LC3

Immunofluorescence multiple-labeled staining was carried out using a TSA-Kit (PerkinElmer Life Sciences, USA). Firstly, the sections were incubated with the first antibody LC3B overnight at 4°C. Then, the sections were incubated with the HRP labeled secondary antibody for 50 min after cleaning. At last, the sections were incubated with Cy3-TSA for 10 min after cleaning. Antigen retrieval was performed by heating in a microwave. Secondly, the sections were incubated with the second antibody p-JNK overnight at 4°C. Then, the sections were again incubated with the HRP labeled secondary antibody for 50 min after cleaning. At last, the sections were incubated with 647-TSA for 10 min after cleaning. Antigen retrieval was again performed by heating in a microwave. Thirdly, the sections were incubated with the third antibody CD8 overnight at 4°C. Then, the sections were again incubated with the FITC-labeled fluorescent secondary antibody for 50 min after cleaning. Finally, the sections were incubated with DAPI (1:500 dilution; Vector Laboratories, CA, USA), and observed under fluorescence microscope.

### Western blotting and immunoprecipitation

Proteins were extracted from Jurkat T cells and CD8^+^ T cells isolated from human, rat peripheral blood and MLR, and protein concentration was measured by BCA method. Equal concentrations of protein were separated by 12% sodium dodecyl sulfate-polyacrylamide gel electrophoresis and transferred onto polyvinylidene difluoride membranes (Millipore, Bedford, MA). The membranes were incubated with rabbit anti-human JNK, p-JNK, Beclin-1, LC3B, P62, GAPDH and rabbit anti-rat JNK, p-JNK, Beclin-1, LC3B, P62, GAPDH antibodies at 4°C overnight, followed by incubation with corresponding secondary antibodies for 1 h. Signal detection and results were recorded using a Tanon-5200CE Chemiluminescent Imaging System (Tanon Science and Technology, China). WB analysis was performed using ImageJ software. For the Beclin-1/Bcl-2 co-immunoprecipitation, Briefly, The lysed cells were pre-cleared with 30μl Protein G Agarose beads (Santa Cruz Biotechnology,USA) for 1 h and incubated with Bcl-2 antibody or beclin-1 antibody at 4°C overnight, followed by adding Protein G Agarose beads and incubating at shaker for 3 h at 4°C. Immunocomplexes were washed 5 times, resuspended in 3x SDS-PAGE loading buffer, boiled for 10 min and subjected to immunoblotting for Beclin-1 and Bcl-2.

### Flow cytometry analysis

PBMCs were isolated from the peripheral blood of patients and rat recipients by Ficoll density-gradient centrifugation according to the manufacturer’s instructions. Cell surface antigens (Ags) were characterized using a standard staining method and measured by flow cytometry. The surface markers of the cells were stained with mAbs against CD3, CD4, CD8. The proliferation of Jurkat T cells was measured by flow cytometric measurement of Ki-67 (BioLegend, USA). An apoptosis detection kit (Beyotime, China) was used for annexin V and PI staining. Dihydroethidium (DHE) (KeyGEN BioTECH, China) fluorescent probe for cytosolic reactive oxygen species (ROS) detection. Moreover, flow cytometry was used to assay for changes in LC3B and p-JNK levels in CD8^+^ T cell. Briefly, the collected cells were resuspended in PBS, then incubated with anti-LC3B and p-JNK primary antibody for 1 h, rinsed with PBS, incubated with fluorescent-conjugated secondary antibodies at room temperature for 30 min. The stained cells were detected by flow cytometry. The results were analyzed by flow cytometry.

### Transmission electron microscopy

TEM is used for the detection of autophagosomes. Liver tissues were fixed with 2.5% glutaraldehyde at pH 7.4, 0.1 M sodium cacodylate for 2–4 h at 44°C, post-fixed, dehydrated, embedded, cut, and stained. Finally, the images were captured using an HT7700 TEM (HITACHI, Japan). Ultrastructural assessment was performed by a blinded observer, and at least three randomly selected areas were evaluated.

### Statistical analysis

All measurements were carried out, at least, in triplicate, and the results are expressed as the mean ± SD. values. Statistical differences between multiple groups were compared using one-way analysis of variance. Statistical differences between two groups were determined using two-tailed unpaired Student’s *t*-test. Analyses were performed with SPSS 26.0. *p*< 0.05 was considered.

## Results

### JNK signaling is involved in autophagy of CD8^+^ T cells during AR in patients

**Figure 1:**
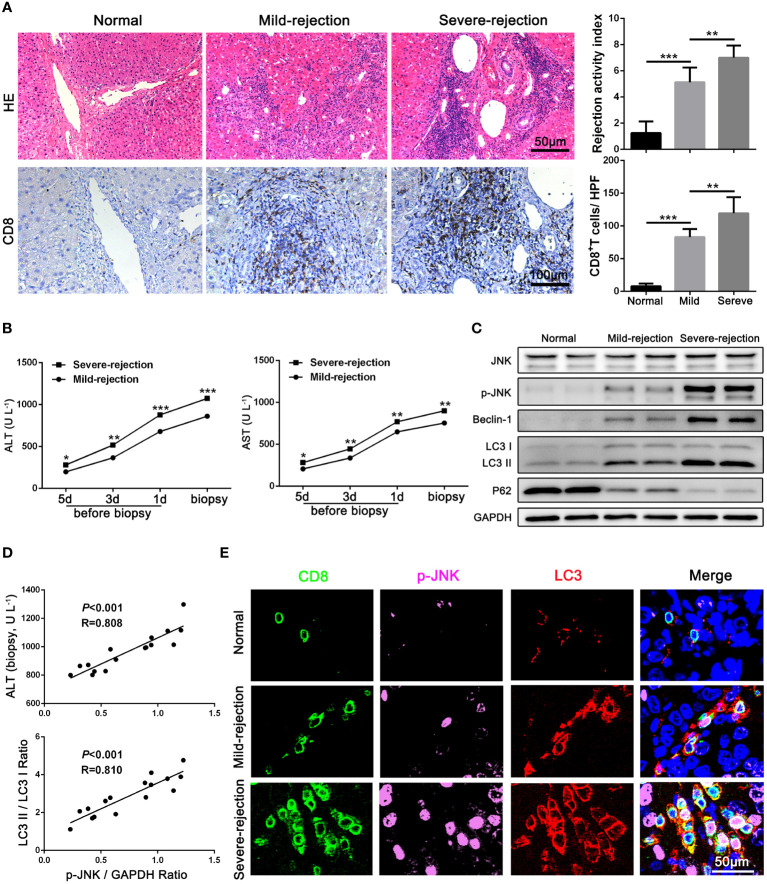
JNK signaling is involved in autophagy of CD8^+^ T cells during AR in patients **(A)** Histopathology (H&E staining) and Immunohistochemical staining of CD8 expression in biopsies with different grades of rejection after LT. The rejection activity index calculated by the Banff scheme and quantification of CD8^+^ T cells were quantified as in **(A)**, right (n=8). **(B)** ALT and AST values in both mild-rejection (n=8) and severe-rejection (n=8) groups in AR patients. **(C)** The protein levels of JNK, p-JNK, Beclin-1, LC3 and P62 in CD8^+^ T cells of peripheral blood isolated from patients were detected by Western Blotting. **(D)** The ratio of p-JNK/GAPDH correlated positively with ALT on biopsy day and with the ratio of LC3 II/LC3 I (n=8). **(E)** Immunofluorescence staining of CD8 (green fluorescence), p-JNK (pink fluorescence) and LC3 (red fluorescence) in biopsies with different grades of rejection after LT. Nuclei were counterstained with DAPI (blue) (n=8). The control sections with normal liver histology were from hepatic hemangioma patients. Each experiment was independently performed more than twice. *p <0.05, **p <0.01, and ***p <0.001; LT, liver transplantation.

To determine the expression of JNK signaling and its relationship with autophagy in CD8^+^ T cells during AR, liver tissues from LT patients (n = 16) and control individuals (n = 10) were collected. Consistent with our previous study, the CD8^+^ T cells infiltrated in liver grafts in the AR group was evidently higher compared with normal liver by IHC staining, and the infiltration of CD8^+^ T cells increased significantly with the aggravation of the rejection (**Figure 1A**). The liver enzymes were significantly higher in patients with severe rejection at the time of initial diagnosis (**Figure 1B**). Then CD8^+^ T cells of peripheral blood were isolated from patients. WB was performed to examine the protein levels of autophagy related proteins and JNK signaling. The results showed that autophagy levels of CD8^+^ T cells increased significantly in severe rejection patients than mild rejection counterparts, as demonstrated by up-regulated autophagy-related proteins LC3 and Beclin-1 levels and down-regulated P62 levels. Similar to the autophagy level, the phosphorylation level of JNK was also significantly higher in the severe rejection group than in the mild rejection group, suggesting the association between the two (**Figure 1C**). And the p-JNK expression was positively correlated with liver function (ALT) and autophagy level (LC3 II/LC3 I ratio) (**Figure 1D**). The distribution of LC3 and p-JNK were further determined by IHC. LC3 and p-JNK were strongly expressed in stromal cells, and both of them are positively correlated with the severity of acute rejection (**Supplementary Figure 1C**). Besides, the multi-label fluorescence staining found that most of p-JNK and LC3 were co-expressed on CD8 positive cell, and their expression increased with the aggravation of rejection (**Figure 1E**). Thus, these data indicates that JNK signaling may involve in CD8^+^ T cell autophagy during AR.

### Activation of JNK positive correlates with the autophagy level of CD8^+^ T cells in rat AR model

**Figure 2:**
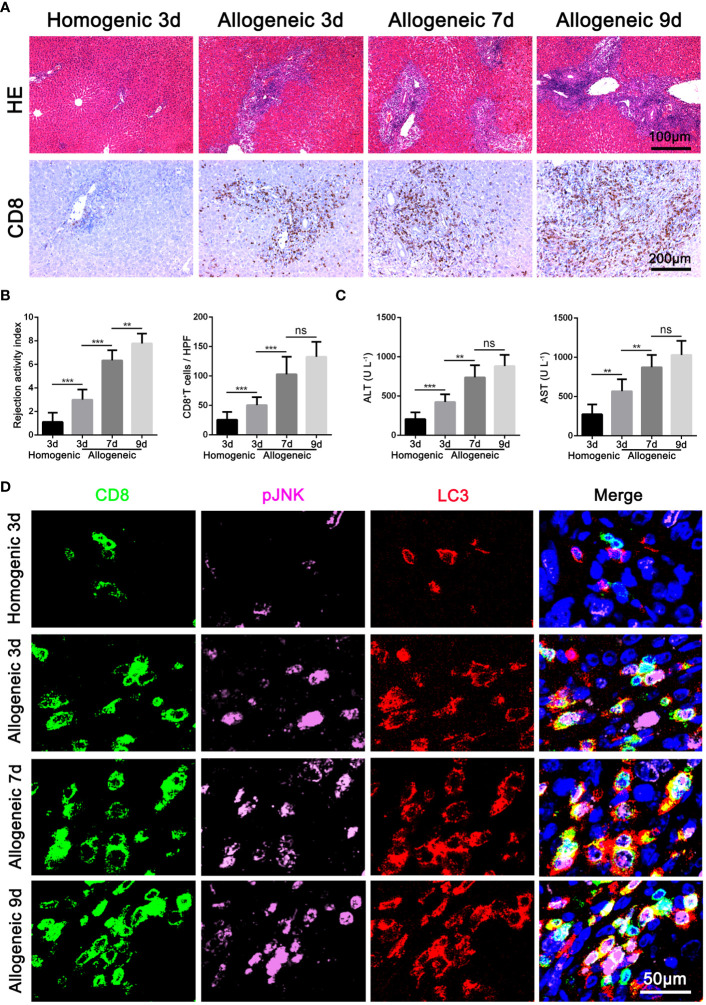
Activation of JNK positive correlates with the autophagy level of CD8^+^ T cells in rat AR model **(A)** Histopathology (H&E staining) and Immunohistochemical staining of CD8 expression in rat AR model at different times after transplantation (n=9). **(B)** The rejection activity index calculated by the Banff scheme and quantification of CD8^+^ T cells were quantified (n=9). **(C)** ALT and AST in serum in rat AR model at different times after transplantation (n=9). **(D)** Immunofluorescence staining of CD8 (green fluorescence), p-JNK (pink fluorescence) and LC3 (red fluorescence) in the liver grafts from rat AR model at different times after transplantation. Nuclei were counterstained with DAPI (blue) (n=9). Each experiment was independently performed more than twice. **p <0.01, and ***p <0.001; AR, acute rejection.

Based on the above results of clinical AR data, we further constructed an acute hepatic rejection model in rats to explore the relation between JNK and autophagy during AR. Compared with homogenic recipients, pathology results showed that AR caused significant inflammatory damage and extensive immune cell infiltration in the liver grafts, accompanied by significantly deteriorated liver function and upregulated inflammatory cytokines. Immunohistochemical results showed that CD8^+^ T cells gradually became the main immune cells infiltrating the graft over time (**Figures 2A-C**, **Supplementary Figure 1D**). In addition, we observed that JNK phosphorylation was significantly enhanced in peripheral blood CD8^+^ T cells from AR rats compared with homogenic recipients and increased with transplantation time by WB, a trend consistent with enhanced autophagy levels (**Supplementary Figure 1B**). Immunohistochemical staining showed that LC3 and p-JNK were highly expressed in mesenchymal cells during liver graft rejection (**Supplementary Figure 1G**). In addition, consistent with the above human LT data, we further confirmed by three-color immunofluorescence that most of p-JNK and LC3 co-color on CD8-positive cells, and the number of three-positive cells was positively correlated with the degree of graft rejection (**Figure 2D**). These data suggest that autophagy levels in CD8^+^ T cells are significantly correlated with JNK phosphorylation during AR of LT in rats.

### Blocking of JNK inhibits CD8^+^ T cell autophagy during AR

**Figure 3:**
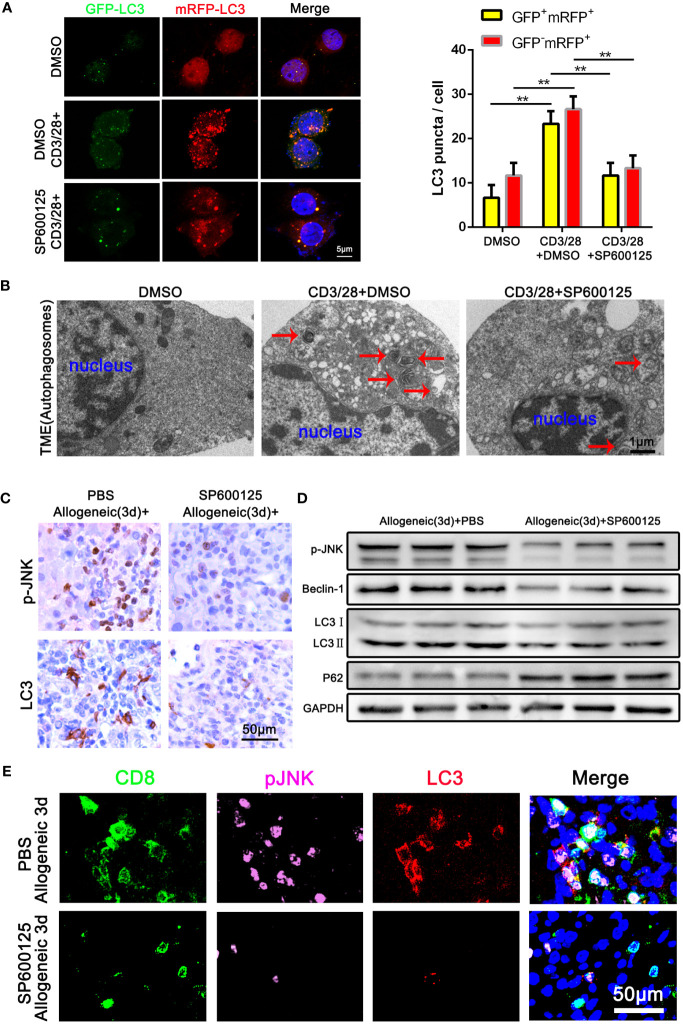
Blocking of JNK inhibits CD8^+^ T cell autophagy during AR **(A)** Jurkat T cells were transfected with StubRFP-sensGFP-LC3 lentivirus and treated with or without SP600125 following the activation by anti-CD3/28 mAbs. Nuclei were counterstained with DAPI (blue). GFP-LC3: green; mRFP-LC3: red; nuclei: blue. The numbers of neutral autophagosomes (GFP^+^mRFP^+^) versus acidified autophagosomes (GFP^−^mRFP^+^) per cell in each condition were quantified as in **(A)**, right. **(B)** Transmission electron microscopy (TEM) images for autophagosome in Jurkat T cells treated with or without SP600125 following the activation by anti-CD3/28 mAbs. The arrow denotes a representative autophagosome. **(C)** Immunohistochemical staining of p-JNK and LC3 in the allogeneic rat recipients on POD3 after LT with or without SP600125 treatment (n=9). **(D)** The protein levels of p-JNK, Beclin-1, LC3 and P62 in CD8^+^ T cells isolated from allogeneic rat recipients’ peripheral blood on POD3 after LT at SP600125 treatment (n=9). **(E)** Immunofluorescence staining of CD8 (green fluorescence), p-JNK (pink fluorescence) and LC3 (red fluorescence) in the allogeneic rat recipients on POD3 after LT with or without SP600125 treatment. Nuclei were counterstained with DAPI (blue) (n=9). Each experiment was independently performed more than twice **p <0.01. AR, acute rejection; LT, liver transplantation; POD, postoperative day. SP600125, a specific JNK inhibitor.

To determine the role of JNK signaling in CD8^+^ T cell autophagy, we first examined the autophagy level of Jurkat T cells treated with JNK inhibitor SP600125 following the activation by anti-CD3/28 mAbs. The autophagy level of activated Jurkat cells were significantly upregulated, which was significantly restrained when JNK signal was inhibited with SP600125 (**Figure 3A**). Transmission electron microscopy (TEM) pictures further revealed an exact reduction of autophagosomes in activated Jurkat T cells treated with SP600125 (**Figure 3B**). We then further established one-way MLR model to mimic acute rejection. The flow cytometry results showed that SP600125 treatment decreased the autophagy level of CD8^+^ T cells (**Supplementary Figure 2A**). Additionally, CD8^+^ T cells subjected to MLR were isolated and the WB analysis revealed that SP600125 treatment significantly decreased autophagy related protein LC3 and Beclin-1, while increased the expression of P62 (**Supplementary Figure 1H**). Thus, these results suggest that JNK blocking inhibited the autophagy level of activated CD8^+^ T cell *in vitro*. To explore the effect of JNK on autophagy in CD8+ T cells during AR *in vivo*, we then further pretreated allogeneic rat recipients with SP600125 by intraperitoneal injection. Samples of blood and liver grafts were collected on POD3 after LT. Immunohistochemical staining and WB showed that SP600125 significantly inhibited JNK activation and reduced autophagy level in liver grafts (**Figures 3C, D**). Triple-color immunofluorescence staining found that CD8 and LC3 double positive cells were significantly reduced after SP600125 treatment (**Figure 3E**). In order to verify whether the regulation of JNK on autophagy depended on the mTOR pathway, our Western blot results showed that compared with the JNK inhibitor SP600125 pretreatment group, the expression of mTOR and phosphorylated mTOR (p-mTOR) in the non-pretreatment group did not change significantly, indicating that JNK regulation of autophagy is independent of mTOR pathway (**Supplementary Figure 3A, B**). Taken together, these data indicate that blocking of JNK signaling inhibits CD8^+^ T cell autophagy during AR both *in vitro* and *in vivo*.

### Blocking of JNK attenuates AR injury by promoting the apoptosis of CD8^+^T cells in rats

**Figure 4:**
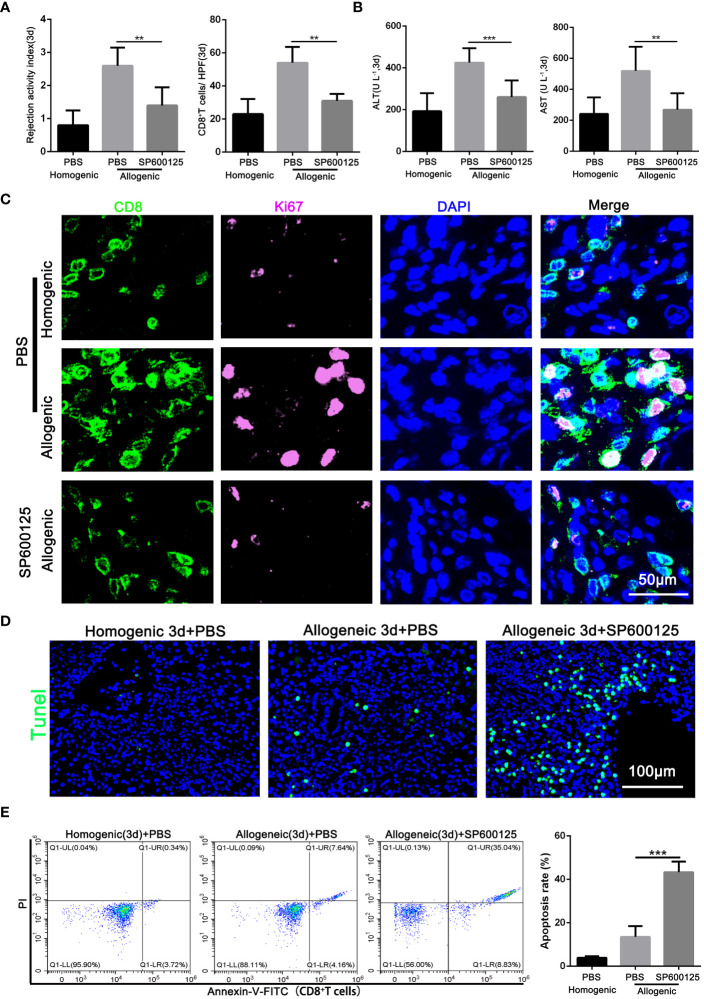
Blocking of JNK attenuates AR injury by promoting the apoptosis of CD8^+^T cells in rats **(A)** Histopathology (H&E staining) and Immunohistochemical staining of CD8 expression in rat AR model on POD3 with or without SP600125 treatment. The rejection activity index calculated by the Banff scheme and quantification of CD8^+^ T cells were quantified (n=9). **(B)** ALT and AST in serum in rat AR model on POD3 with or without SP600125 treatment (n=9). **(C)** Immunofluorescence staining of CD8 (green fluorescence) and Ki-67 (pink fluorescence) in the liver grafts from rat AR model on POD3 at SP600125 treatment. Nuclei were counterstained with DAPI (blue) (n=9). **(D)** TUNEL (green) assisted detection of hepatic apoptosis in rat AR model of homogenic and allogeneic rat at SP600125 treatment (n=9). **(E)** The percentage of apoptotic CD8^+^ T cells isolated from rat AR model on POD3 at SP600125 treatment was detected by flow cytometry (n=9). Each experiment was independently performed more than twice. **p <0.01 and ***p <0.001. AR, acute rejection; POD, postoperative day. SP600125, a specific JNK inhibitor.

After confirming that JNK affects the autophagy of CD8^+^ T cells, the effect of JNK inhibition on AR injury was further explored. Samples of blood and liver grafts from SP600125 pretreated recipients were collected on POD3. As shown in **Figure 4A** and **Supplementary Figure 1F**, histological analysis showed that SP600125-treated recipients had less liver rejection damage compared with untreated rats, as well as significantly fewer infiltrated CD8^+^ T cells, as measured by the number of CD8-positive cells in the grafts stained by immunohistochemistry. These changes were accompanied by an increase of liver enzymes in serum and inflammatory cytokines in serum and liver grafts (**Figure 4B**, **Supplementary Figure 1E**). The above results indicates that JNK inhibition had a comprehensive protective effect on the graft.

Our previous studies showed that inhibition of autophagy undermines CD8^+^ T cell proliferation, and then we further investigated whether JNK inhibition was also related to the proliferation level of CD8^+^ T cells. Immunofluorescence staining confirmed that SP600125 administration significantly reduced the number of Ki-67-positive CD8^+^ T cells infiltrating the graft (**Figure 4C**). Additionally, given the closely link between JNK and apoptosis, we further investigated whether JNK inhibition affect the survival rate of CD8^+^ T cells during AR. TUNEL staining showed that the number of apoptotic infiltrating immune cells increased significantly after the application of JNK inhibitor (**Figure 4D**). Flow cytometry results further confirmed that JNK inhibitor administration significantly increased the apoptosis rate of peripheral blood CD8^+^ T cells in allogenic rat recipients (**Figure 4E**). In addition, Mitochondrial stability is essential for cell survival. FACS analysis revealed that compared to untreated rats, the treatment of JNK inhibitor also significantly increased the level of mitochondrial ROS in peripheral blood CD8^+^ T cells from allogenic recipient rats (**Supplementary Figure 2B**). Moreover, the functional evaluation of CD8^+^ T cells showed that inhibition of JNK significantly reduced the cytotoxicity of CD8^+^ T cells by decreasing the expression of CD107 and granzyme B (**Supplementary Figure 2C, D**). These results suggest that JNK inhibition promotes the apoptosis of CD8^+^ T cells during acute rejection and may be achieved by disrupting mitochondrial stability by increasing ROS.

### Pro-survival effect of JNK on activated T cells depends on activating autophagy

**Figure 5:**
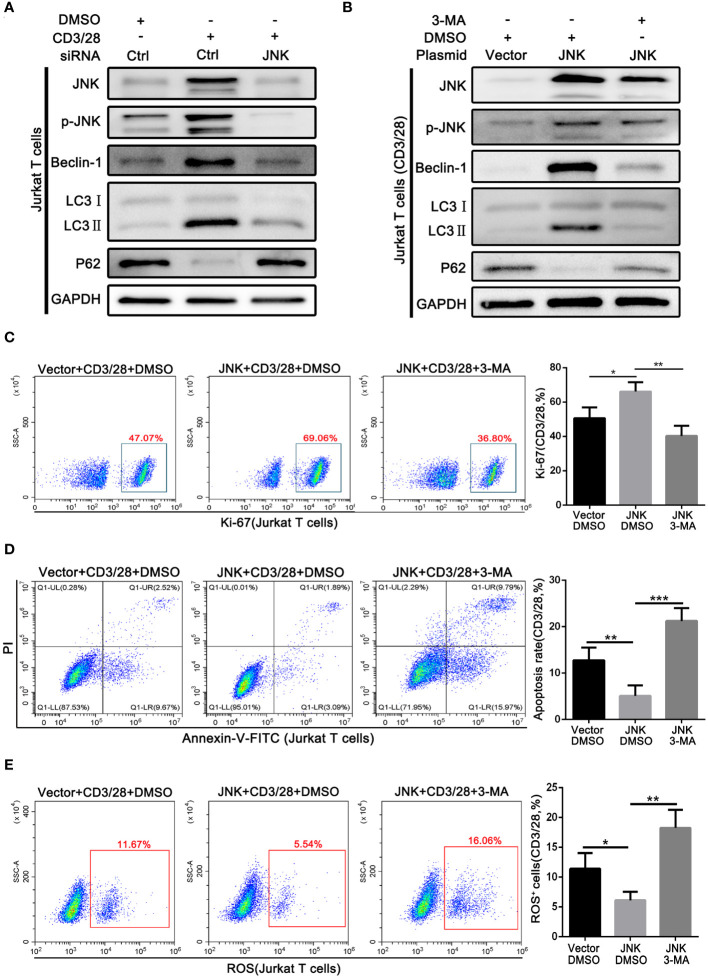
Pro-survival effect of JNK on activated T cells depends on activating autophagy **(A)** The protein levels of p-JNK, Beclin-1, LC3 and P62 in Jurkat T cells following transfection by JNK-siRNA and activation by anti-CD3/28 mAbs at SP600125 treatment. **(B)** The protein levels of p-JNK, Beclin-1, LC3 and P62 in Jurkat T cells following transfection by JNK-overexpressing plasmid and activation by anti-CD3/28 mAbs at 3-MA treatment. **(C)** Flow cytometric analysis of Ki-67 in Jurkat T cells following transfection by JNK-overexpressing plasmid and activation by anti-CD3/28 mAbs at 3-MA treatment. Quantitative analysis of flow cytometric data was shown as in **(C)**, right. **(D)** The percentage of apoptotic Jurkat T cells following transfection by JNK-overexpressing plasmid and activation by anti-CD3/28 mAbs at 3-MA treatment. Quantitative analysis of flow cytometric data was shown as in **(D)**, right. **(E)** ROS staining by flow cytometry in Jurkat T cells following transfection by JNK-overexpressing plasmid and activation by anti-CD3/28 mAbs at 3-MA treatment. Quantification of ROS-positive cells were quantified as in **(E)**, right. Each experiment was independently performed more than twice. *p <0.05, **p <0.01, and ***p <0.001. 3-MA, 3-methyladenine, a specific autophagy inhibitor.

On the basis of the above findings, we further explored whether the protective effect of JNK on CD8^+^ T cells in acute rejection depends on the activation of autophagy. Subsequently, Jurkat T cell lines were transfected with JNK plasmid and siRNA to precisely regulate the expression of JNK signaling, followed by costimulatory activation with CD3/28. Endogenous knockdown of JNK significantly reduced the elevation of co-stimulation induced autophagy activation, suggesting the importance of JNK signaling in the regulation of autophagy during T-cell activation (**Figure 5A**). In contrast, overexpression of JNK further increased the autophagy level of CD3/28-stimulated cells compared with the control group (**Figure 5B**). Furthermore, after transfection of JNK plasmid, we treated Jurkat T cell line with autophagy inhibitor 3-MA, and found that autophagy was fully inhibited, Ki-67 expression was significantly reduced (**Figure 5C**). And inhibition of autophagy on the basis of overexpression of JNK significantly increased the apoptosis of Jurkat T cells (**Figure 5D**). Moreover, Mitochondrial stability is essential for cell survival. FACS analysis revealed that compared to untreated Jurkat T cells, the treatment of autophagy inhibitor also significantly increased the level of mitochondrial ROS (**Figure 5E**). These results suggest that the protective effect of JNK on activated T cells was dependent on autophagy.

### JNK may activate autophagy in CD8^+^ T cells by inhibiting the formation of Bcl-2/BECN1 complex

**Figure 6:**
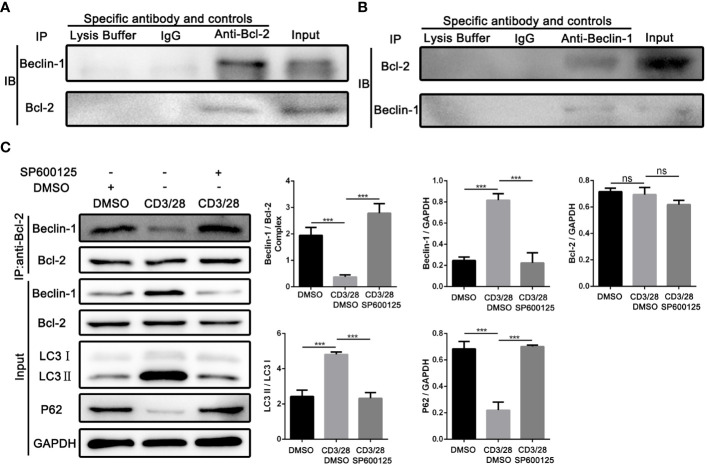
JNK may activate autophagy in CD8^+^ T cells by inhibiting the formation of Bcl-2/BECN1 complex **(A)** Total lysates from co-stimulation activated Jurkat T cells were immunoprecipitated with anti-Bcl-2 antibody, or normal IgG or lysate buffer followed by immunoblotted with BECN1 and Bcl-2 antibodies respectively. The far-right line was immunoblotted for BECN1 and Bcl-2 protein in input proteins. **(B)** Total lysates from co-stimulation activated Jurkat T cells were immunoprecipitated with anti-BECN1 antibody, or normal IgG or lysate buffer followed by immunoblotted with BECN1 and Bcl-2 antibodies respectively. The far-right line was immunoblotted for BECN1 and Bcl-2 protein in input proteins. **(C)** Immunoblots of lysates from Jurkat T cells treated with SP600125 following the activation by anti-CD3/28 mAbs. The relative Beclin-1, Bcl-2, LC3 and P62 expression levels in Jurkat T cells were detected by Western Blotting as in **(C)**, right. Each experiment was independently performed more than twice. ***p <0.001; ns, no significance, a specific JNK inhibitor.

Next, we investigated the underlying mechanism of JNK in regulating the autophagy of CD8^+^ T cells during AR. According to the above results, we found that Beclin1 was significantly up-regulated in CD8^+^ T cells from clinical rejection liver tissues and allogenic rat grafts, while JNK inhibition significantly down-regulated Beclin1 expression. Beclin1 is a key factor in the early induction of autophagy, and JNK activation has been reported to promote dissociation between Bcl-2 and BECN1, thereby upregulating Beclin1. We hypothesized that activated JNK signaling may affect CD8^+^ T cell autophagy by regulating the formation of Bcl-2/BECN1 complex. To test this hypothesis, co-stimulation activated Jurkat cells with or without SP600125 treatment were collected and used for immunoprecipitation with antibodies against Bcl-2. When the corresponding IP fraction was immunostained with anti-Bcl-2, a single band of approximately 26kDa was observed, indicating that Bcl-2 was successfully immunoprecipitated (**Figures 6A, B**). Western blot analysis of the IP content revealed that BECN1 was co-precipitated by Bcl-2 antibodies, implying a physical interaction between Bcl-2 and BECN1. However, the binding affinity of BECN1 to Bcl-2 was significantly inhibited after costimulatory activation. In contrast, SP600125 partially restored Bcl-2/BECN1 complex formation under activation conditions (**Figure 6C**). Therefore, the formation of Bcl-2/BECN1 complex may be an important step in the activation of autophagy in T cells by JNK signaling pathway.

### JNK mediates acute rejection via activating autophagy of CD8^+^ T cells through upregulating BECN1

**Figure 7:**
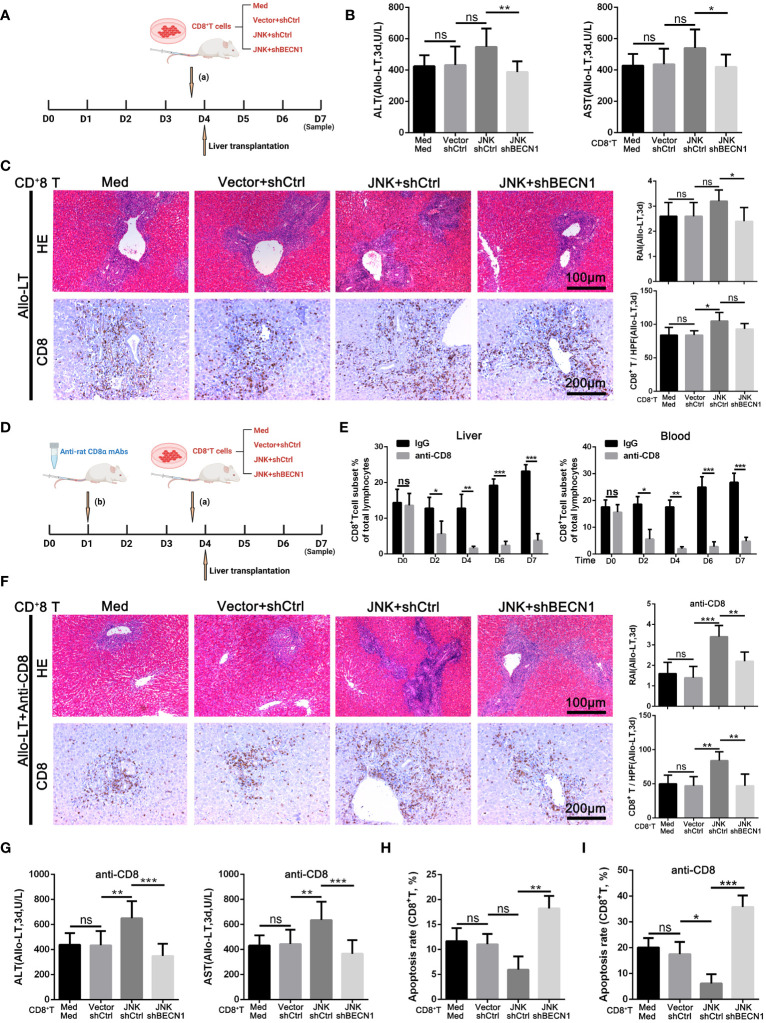
JNK mediates acute rejection via activating autophagy of CD8^+^ T cells through upregulating BECN1 **(A–C)** Splenic derived CD8^+^ T cells (2 × 10^6^/L) from LW rats pretreated with med, JNK overexpression or BECN1 knockdown after JNK overexpression were transfused into BN recipient rats on the day before transplantation. Specimens were obtained three days after surgery. **(A)** Scheme illustrating experimental set-up. **(B)** Analysis of serum levels of ALT and AST between groups in rat AR model (n=9). **(C)** Histopathology (H&E staining) and Immunohistochemical staining of CD8 expression in liver tissues of rats between groups in rat AR model. The rejection activity index calculated by the Banff scheme and quantification of CD8^+^ T cells were quantified (n=9). **(D–G)** Splenic derived CD8^+^ T cells (2 × 10^6^/L) from LW rats with the same pretreatments as above were transfused into BN recipient rats on the day before transplantation after anti rat CD8 antibodies administration 3 days before. **(D)** Scheme illustrating experimental set-up. **(E)** Flow cytometric analysis of the percentage of CD4^+^ and CD8^+^ T cells in the rat recipients’ peripheral blood and liver tissue injected with anti-rat CD8 antibody during peri-liver transplantation. **(F)** Histopathology (H&E staining) and Immunohistochemical staining of CD8 expression in liver tissues of rats between groups in rat AR model. The rejection activity index calculated by the Banff scheme and quantification of CD8^+^ T cells were quantified (n=9). **(G)** Analysis of serum levels of ALT and AST between groups in rat AR model (n=9). **(H, I)** Percentage of apoptotic CD8^+^ T cells isolated from rat injected with different plasmid CD8^+^ T cells (n=9). Each experiment was independently performed more than twice. *p <0.05, **p <0.01, and ***p <0.001; ns, no significance.; Allo-LT, allogenic liver transplantation.

**Figure 8:**
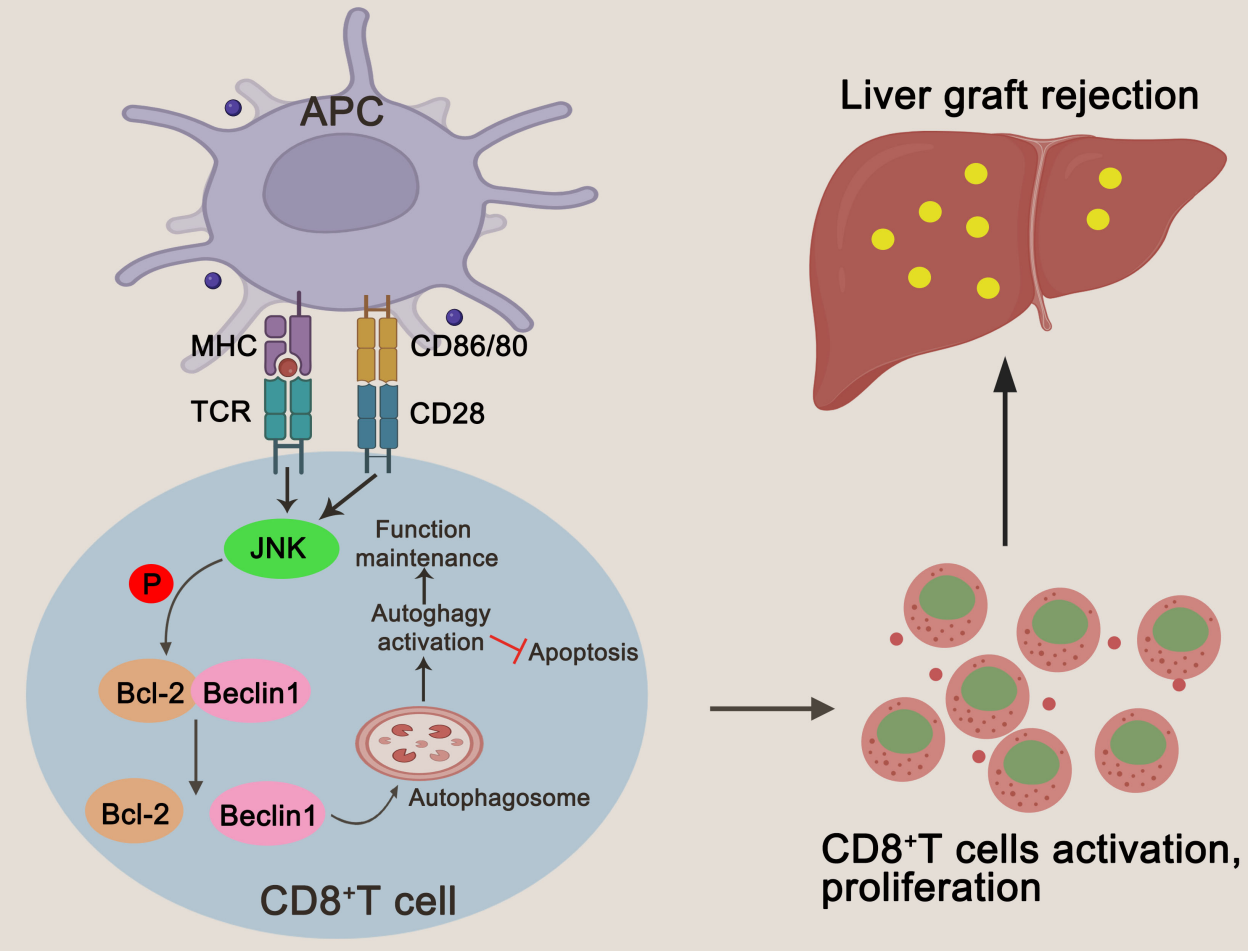
A schematic representation of JNK regulating autophagy in T cells by up-regulating BECN1 by inhibiting the formation of the Bcl-2/BECN1 complex. Drawing created with BioRender.

Based on the above experiment, JNK inhibition inhibits CD8^+^ T cell autophagy *in vitro* by regulating BECN1, thereby promoting apoptosis. In order to further clarify that JNK inhibition attenuates rejection injury by regulating BECN1 in rat models, splenic derived CD8^+^ T cells with BECN1 knockdown based on JNK overexpression were transfused into recipient rats the day before transplantation, and specimens were obtained three days after surgery (**Figure 7A**). In line with the *in vitro* results, the infusion of CD8^+^ T cells overexpressing JNK aggravated liver transplant rejection injury, while BENC1 knockdown reduced this effect (**Figures 7B, C**). Flow cytometry analysis also showed that the apoptosis level of CD8^+^ T cells decreased after the infusion of CD8^+^ T cells with JNK overexpression, and significantly increased after infusion of BECN1 knockdown lines on the basis of JNK overexpression (**Figure 7H**, **Supplementary Figure 3C**). In view of the interfering effect of the recipient rat’s own CD8^+^ T cells, we used anti-rat CD8 antibody to block the recipient rat’s own CD8^+^ T cells 3 days before liver transplantation (**Figure 7D**). Flow cytometry analysis showed that the proportion of CD8^+^T cells in both peripheral blood and liver tissue were at a low level before LT, and remained at a low level for 3 days after LT (**Figure 7E**). After blocking rats’ own CD8^+^ T cells in advance, infusion of BECN1 knockdown lines on the basis of JNK overexpression significantly reduced JNK-mediated transplantation rejection damage and increased the apoptosis level of CD8^+^ T cells (**Figures 7F-G, I**, **Supplementary Figure 3D**). These results further confirmed that JNK signaling up-regulates BECN1-promoted autophagy mediated AR after LT both *in vivo* and *in vitro* (**Figure 8**).

## Discussion

Hepatic acute rejection after transplantation is an inflammatory disease characterized by persistent lymphatic immune damage to liver grafts. Potent immunosuppressive agents obviously but do not completely reduce the incidence of acute rejection, and so far, the pathogenesis of acute rejection has been not fully elucidated. Our previous studies have shown that autophagy is critical for maintaining the survival of activated CD8^+^ T cells during acute rejection, and inhibition of autophagy significantly attenuates rejection by promoting CD8^+^ T cell apoptosis (21–23). Here, we further explored the mechanism of mediating autophagy of CD8^+^ T cells during acute rejection and found that JNK signaling is involved in the activation of autophagy of CD8^+^ T cells. Inhibition of JNK signaling can attenuate rejection by inhibiting autophagy of CD8^+^ T cells and promoting their apoptosis. More importantly, we preliminary revealed that JNK regulation of autophagy in T cells may be mediated by up-regulation of BECN1 through inhibition of Bcl-2/BECN1 complex formation.

T cell mediated rejection is the main form of acute rejection after transplantation. Previous studies pointed that CD4^+^ T cells are the critical driver of graft rejection, which cause graft injury directly through Fas-Fas ligand-mediated apoptosis, or indirectly attract macrophages through producing inflammatory cytokines and help activation of cytotoxic CD8^+^ T cells (20, 22, 24). Recently, the role of CD8^+^ T cell in graft rejection has been paid more attention due to its potent ability to directly kill graft parenchymal cells and vascular endothelial cells. Su et al. (25) in the study of mice heart transplantation model found the important role of endogenous memory CD8^+^ T cells in directly mediating allograft injury and demonstrated that these T cells could directly trigger graft failure in mice, and inhibition of endogenous memory CD8^+^ T cell graft infiltration attenuated this injury and prolonged graft survival. Balam et al. (26) also demonstrated that depletion of CD4^+^ T cells in recipient mice did not prevent the progression of cardiac rejection, and CD8^+^ T cells are critical for mediating chronic rejection. Similarly, in this study and in our previous studies (9, 20), we found that the liver grafts of patients with acute rejection were infiltrated with a large number of CD8^+^ T cells. And in the rat hepatic acute rejection model, CD8^+^ T cells gradually become the main immune cells with the progress of rejection. In addition, reducing CD8^+^ T cells by inhibition of autophagy or JNK signaling can significantly attenuates rejection damage after LT in rats. These data confirm the critical role of CD8^+^ T cells in acute rejection and highlight their importance as potential targets in immunotolerance therapies.

JNK is a serine-threonine protein kinase belonging to the MAPK family, which is involved in the regulation of cell proliferation, differentiation and apoptosis. Several studies have shown that JNK is involved in the activation and proliferation of T cells and is involved in the rejection after transplantation. Under T cell receptor (TCR)/CD28 costimulation, MAPK4/7 are activated, and activated MAPK4/7 are further phosphorylated to activate JNK, thereby regulating cell survival, differentiation and proliferation (16, 27, 28). Tabata et al. (29) showed that JNK inhibitor SP600125 could effectively inhibit T cell-mediated alloimmune response and thus prolong the survival time of rat cardiac grafts. Chen et al. (17) found that JNK inhibition significantly inhibited the mixed lymphocyte response and T lymphocyte proliferation *in vitro*, as well as T cell-mediated alloimmune response *in vivo* in a rat kidney transplantation model. Consistently, in the present study, we found significant activation of JNK in CD8^+^ T cells in both LT patients and rats with acute rejection, suggesting that JNK may be involved in acute rejection. Interestingly, we also found a clear positive correlation between JNK phosphorylation and autophagy activation. Given that our previous studies have demonstrated the importance of autophagy for the survival of activated CD8^+^ T cells during acute rejection, we hypothesized that JNK may participate in acute rejection by activating autophagy of CD8^+^ T cells. To test this mechanism, we first used SP600125, a specific inhibitor that inhibits JNK signaling activation both *in vivo* and *in vitro*. The results showed that SP600125 treatment significantly down-regulated the autophagy level of activated Jurkat cells and CD8^+^ T cells of acute rejection rats. Besides, SP600125 treatment also reduced the proliferation level and promoted apoptosis of CD8^+^ T cells in recipient rats, thereby improved graft rejection injury. Furthermore, activated Jurkat cells were overexpressed JNK *in vitro* and treated with autophagy inhibitor 3-MA. We found that the protective effect of JNK activation on cell survival was subside after autophagy inhibition, which preliminarily confirmed that the effect of JNK inhibition on the number of CD8^+^ T cells was dependent on the reduction of the autophagy level of CD8^+^ T cells. To further investigate the effect of direct modulation of JNK signaling in CD8^+^ T cells on graft rejection in a rat model, we injected CD8^+^ T cells transfected with JNK plasmid overexpression into BN rats through tail vein. The results showed that CD8^+^ T cells overexpressing JNK significantly increased the rejection injury after liver transplantation.

The mechanism by which JNK regulates autophagy in activated CD8^+^ T cells during acute rejection was also explored in this study. We found that Beclin1 expression was significantly up-regulated in both CD8^+^ T cells from patients with acute rejection and allogeneic recipient rats, and activated Jurkat cells. As one of the key factors in the early activation of autophagy, Beclin1 initiates autophagy by recruiting autophagy/endosomal regulatory proteins such as PIK3C3, UVRAG and ATG14 (30–32). As with Beclin-1, Bcl-2 contains BH domains, which can affect the activity of BH3 by binding to Beclin-1 to form a complex. Increasing the Beclin-1/Bcl-2 complex leads to a decrease in autophagy. Beclin-1 initiates autophagy when the Beclin-1/Bcl-2 complex dissociates, whereas Bcl-2 inhibits apoptosis (33–35). JNK is involved in the regulation of different pathways of autophagy. JNK signal has been reported to promote the binding of PIK3C3 to BECN1 by inhibiting BECN1 binding to Bcl-2 family members, thereby activating the autophagy process (13). Ke et al. (36) found that JNK1’s pro-osteoclast function was dependent on its phosphorylation of the downstream signal Bcl-2, which further inhibited Bcl-2’s interaction with Beclin1and promoted the autophagy process. Zhang et al. (37) found that autophagy prevents monocyte apoptosis and also induces monocyte-macrophage differentiation, which can be attributed to JNK-mediated Bcl2 phosphorylation and subsequent Beclin1/Bcl2 dissociation. Here, we show that autophagy is associated with the Bcl-2/BECN1 system in activated Jurkat cells *in vitro*. Costimulatory activation upregulated BECN1 expression and increased the level of the free form of BECN1 that had been dissociated from the Bcl-2/BECN1 complex. In contrast, JNK inhibitor SP600125 enhanced the interaction between Bcl-2 and BECN1. This suggests that the elevated expression of BECN1 and its dissociation from Bcl-2 are responsible for JNK-mediated autophagy. Moreover, JNK inhibitor (SP600125) significantly increased the levels of mitochondrial ROS and apoptosis, indicating that Mitochondrial stability plays a crucial role in the regulation of above process. In the rat model, CD8^+^ T cells transfected with plasmid were injected into the tail vein to observe the effect of JNK-mediated autophagy on liver transplantation injury. The results showed that BECN1 knockdown of CD8^+^ T cells could significantly reduce JNK-mediated liver transplantation rejection injury. These results suggest that JNK mediates acute rejection of liver transplantation by up-regulating the dissociated BECN1 in Bcl-2/BECN1 complex to promote autophagy.

There are undoubtedly some limitations to our study. Although we confirmed in this study that inhibition of JNK can reduce acute rejection after liver transplantation by down-regulating autophagy of CD8^+^ T cells and promoting their apoptosis, it is indisputable that other cells such as CD4^+^ T cells, DC cells, and innate immune cells including neutrophils and macrophages can also play important roles in rejection after transplantation. Whether JNK can also influence rejection reaction by regulating the above-mentioned immune cells remains to be determined. Besides, we found that JNK up-regulates T cell autophagy by inhibiting BCL2-BECN1 complex formation through *in vitro* T cell activation model, but the activation of T cells induced by anti-CD3/CD28 is not exactly equivalent to the activation state of T cells during transplantation. The mechanisms by which JNK regulates CD8^+^ T cell autophagy during LT remains to be further elucidated. In addition, we confirmed that inhibition of JNK at a given dose concentration of inhibitor could alleviate liver transplantation rejection in rat models, but the possible damage of JNK inhibitor to hepatic parenchymal cells is noteworthy, and more studies are needed to verify the safety and efficacy of JNK inhibitors before clinical application. However, our study emphasizes the importance of autophagy in acute rejection, and suggests that inhibition of JNK signaling can be used as one of the means to target autophagy regulation, thus providing a new strategy for the treatment of graft rejection.

## Data availability statement

The original contributions presented in the study are included in the article/**Supplementary Material**. Further inquiries can be directed to the corresponding authors.

## Ethics statement

Ethical approval was not required for the studies on humans in accordance with the local legislation and institutional requirements because only commercially available established cell lines were used. The animal study was approved by the ethics committee of the Third Affiliated Hospital of Sun Yat-Sen University. The study was conducted in accordance with the local legislation and institutional requirements.

## Author contributions

XW: Methodology, Writing – original draft, Data curation, Formal analysis, Resources, Software. WZ: Data curation, Formal analysis, Methodology, Resources, Writing – original draft. HC: Formal analysis, Methodology, Resources, Software, Writing – original draft. XL: Data curation, Formal analysis, Investigation, Methodology, Writing – original draft. WZ: Data curation, Formal analysis, Methodology, Resources, Writing – original draft. YZ: Data curation, Formal analysis, Investigation, Methodology, Writing – original draft. NF: Supervision, Validation, Visualization, Writing – review & editing. XC: Funding acquisition, Supervision, Validation, Visualization, Writing – review & editing. GW: Conceptualization, Funding acquisition, Project administration, Supervision, Writing – review & editing.

## Supplementary material

Supplementary Figure 1
**(A)** The percentage of purified CD8^+^ T cells. **(B)** The protein levels of JNK, p-JNK, Beclin-1, LC3 and P62 in CD8+ T cells of peripheral blood isolated from rat recipients at different times after transplantation. **(C)** Immunohistochemical staining of p-JNK and LC3 in biopsies with different grades of rejection after LT (n = 8). **(D)** Serum levels of TNF-α and INF-γ in rat recipients at different times after transplantation (n = 9). **(E)** TNF-α and INF-γ in serum in rat AR model on POD3 with or without SP600125 treatment (n = 9). **(F)** Histopathology (H&E staining) and Immunohistochemical staining of CD8 expression in rat AR model on POD3 with or without SP600125 treatment (n = 9). **(G)** Immunohistochemical staining of p-JNK and LC3 in the liver grafts from rat AR model at different times after transplantation (n = 9). **(H)** The protein levels of p-JNK, Beclin-1, LC3 and P62 in CD8^+^ T cells from one-way MLR model treated with or without SP600125. Each experiment was independently performed more than twice. **P* <0.05, ****P* <0.001 and *****P* <0.0001; ns, no significance; AR, acute rejection; POD, postoperative day; SP600125, a specific JNK inhibitor; MLR, mixed lymphocyte reaction.

Supplementary Figure 2
**(A)** Flow cytometric analysis of p-JNK and LC3 expression in CD8^+^ T cells from one-way MLR model treated with or without SP600125. **(B)** ROS staining by flow cytometry in CD8^+^ T cells isolated from rat AR model on POD3 at SP600125 treatment. **(C)** Representative flow cytometric profiles of CD107a expression in CD8^+^ T cells isolated from rat AR model on POD3 at SP600125 treatment. **(D)** Representative flow cytometric profiles of granzyme B expression in CD8^+^ T cells isolated from rat AR model on POD3 at SP600125 treatment. Each experiment was independently performed more than twice. AR, acute rejection; POD, postoperative day; SP600125, a specific JNK inhibitor; MLR, mixed lymphocyte reaction.

Supplementary Figure 3
**(A)** The protein levels of JNK, mTOR, p-mTOR and Beclin-1 in CD8^+^ T cells isolated from allogeneic rat recipients’ peripheral blood on POD3 after LT at SP600125 treatment (n=9). **(B)** The protein levels of JNK, JNK, mTOR, p-mTOR and Beclin-1 in Jurkat T cells following activation by anti-CD3/28 mAbs at SP600125 treatment. **(C)** BN rats were not injected with anti-rat CD8 antibody through the tail vein three days before liver transplantation. The percentage of apoptotic CD8^+^ T cells isolated from rat injected with different plasmid CD8^+^ T cells (n = 9). **(D)** BN rats were injected with anti-rat CD8 antibody through the tail vein three days before liver transplantation. The percentage of apoptotic CD8^+^T cells isolated from rat injected with different plasmid CD8^+^ T cells (n = 9). Each experiment was independently performed more than twice. Allo-LT, allogenic liver transplantation.
