# Supplementary figures and images for: JNK signaling mediates acute rejection via activating autophagy of CD8+ T cells after liver transplantation in rats
Source: Front Immunol. 2024 Mar 18;15:1359859. doi: 10.3389/fimmu.2024.1359859 (PMC10982410; doi:10.3389/fimmu.2024.1359859)

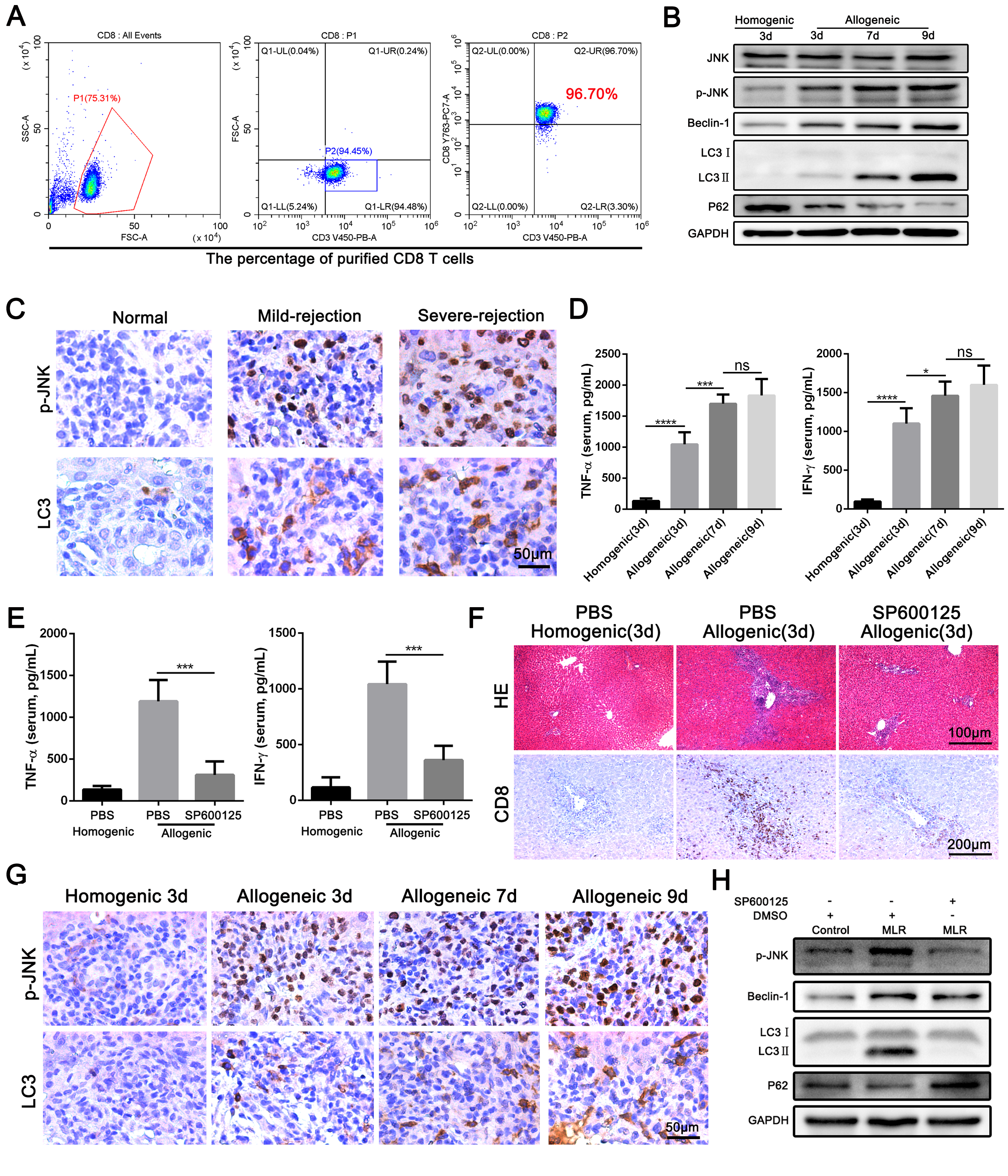

Supplement: Supplementary Figure 1 — (A) The percentage of purified CD8+ T cells. (B) The protein levels of JNK, p-JNK, Beclin-1, LC3 and P62 in CD8+ T cells of peripheral blood isolated from rat recipients at different times after transplantation. (C) Immunohistochemical staining of p-JNK and LC3 in biopsies with different grades of rejection after LT (n = 8). (D) Serum levels of TNF-α and INF-γ in rat recipients at different times after transplantation (n = 9). (E) TNF-α and INF-γ in serum in rat AR model on POD3 with or without SP600125 treatment (n = 9). (F) Histopathology (H&E staining) and Immunohistochemical staining of CD8 expression in rat AR model on POD3 with or without SP600125 treatment (n = 9). (G) Immunohistochemical staining of p-JNK and LC3 in the liver grafts from rat AR model at different times after transplantation (n = 9). (H) The protein levels of p-JNK, Beclin-1, LC3 and P62 in CD8+ T cells from one-way MLR model treated with or without SP600125. Each experiment was independently performed more than twice. *P <0.05, ***P <0.001 and ****P <0.0001; ns, no significance; AR, acute rejection; POD, postoperative day; SP600125, a specific JNK inhibitor; MLR, mixed lymphocyte reaction. [file Image_1.tif]

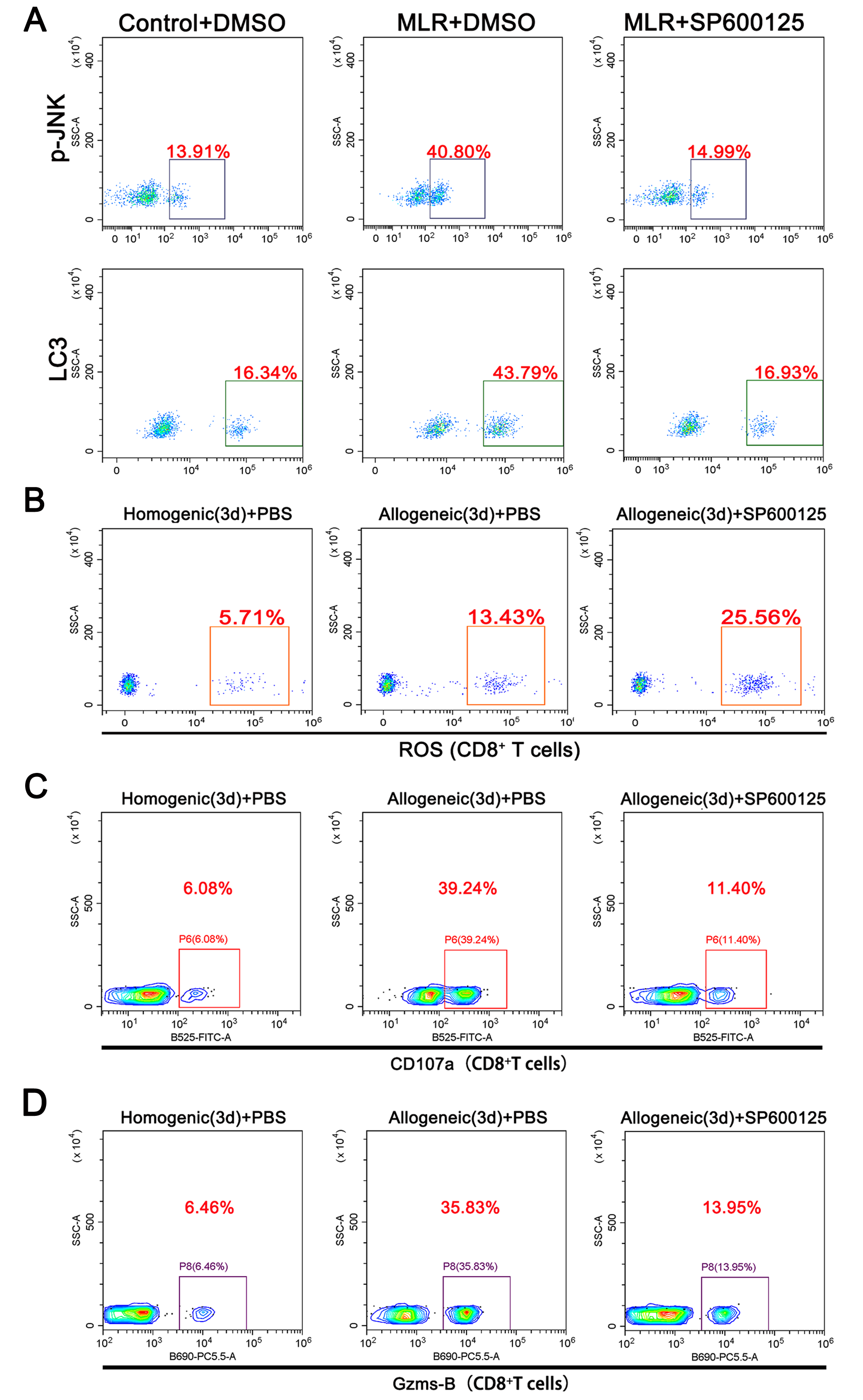

Supplement: Supplementary Figure 2 — (A) Flow cytometric analysis of p-JNK and LC3 expression in CD8+ T cells from one-way MLR model treated with or without SP600125. (B) ROS staining by flow cytometry in CD8+ T cells isolated from rat AR model on POD3 at SP600125 treatment. (C) Representative flow cytometric profiles of CD107a expression in CD8+ T cells isolated from rat AR model on POD3 at SP600125 treatment. (D) Representative flow cytometric profiles of granzyme B expression in CD8+ T cells isolated from rat AR model on POD3 at SP600125 treatment. Each experiment was independently performed more than twice. AR, acute rejection; POD, postoperative day; SP600125, a specific JNK inhibitor; MLR, mixed lymphocyte reaction. [file Image_2.tif]

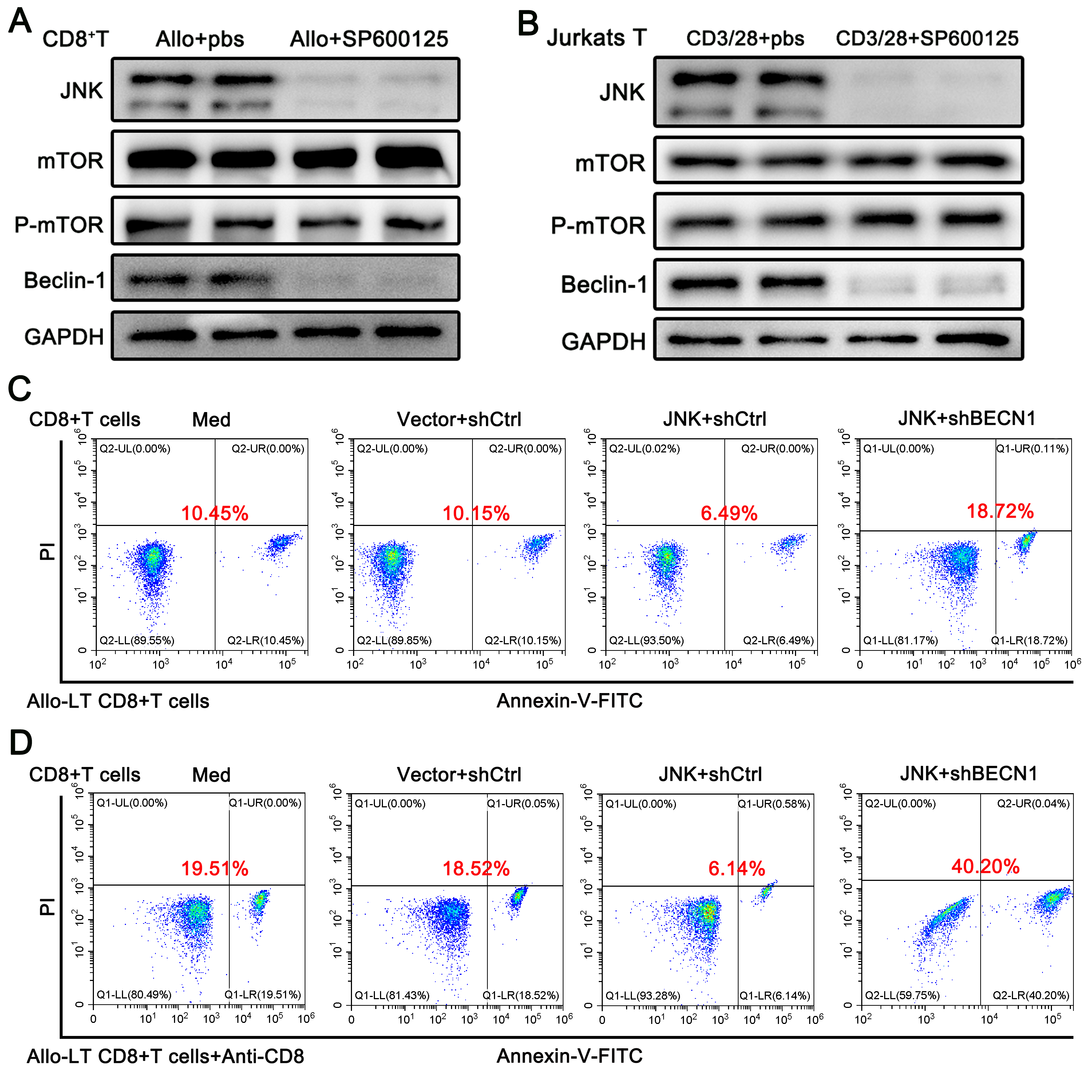

Supplement: Supplementary Figure 3 — (A) The protein levels of JNK, mTOR, p-mTOR and Beclin-1 in CD8+ T cells isolated from allogeneic rat recipients’ peripheral blood on POD3 after LT at SP600125 treatment (n=9). (B) The protein levels of JNK, JNK, mTOR, p-mTOR and Beclin-1 in Jurkat T cells following activation by anti-CD3/28 mAbs at SP600125 treatment. (C) BN rats were not injected with anti-rat CD8 antibody through the tail vein three days before liver transplantation. The percentage of apoptotic CD8+ T cells isolated from rat injected with different plasmid CD8+ T cells (n = 9). (D) BN rats were injected with anti-rat CD8 antibody through the tail vein three days before liver transplantation. The percentage of apoptotic CD8+T cells isolated from rat injected with different plasmid CD8+ T cells (n = 9). Each experiment was independently performed more than twice. Allo-LT, allogenic liver transplantation. [file Image_3.tif]
